# Does chess instruction improve mathematical problem-solving ability? Two experimental studies with an active control group

**DOI:** 10.3758/s13420-017-0280-3

**Published:** 2017-06-23

**Authors:** Giovanni Sala, Fernand Gobet

**Affiliations:** Department of Psychological Sciences, University of Liverpool, Bedford Street South, Liverpool, L69 7ZA UK

**Keywords:** Chess, Expertise, Instruction, Learning, Meta-analysis, Transfer

## Abstract

It has been proposed that playing chess enables children to improve their ability in mathematics. These claims have been recently evaluated in a meta-analysis (Sala & Gobet, 2016, *Educational Research Review, 18,* 46–57), which indicated a significant effect in favor of the groups playing chess. However, the meta-analysis also showed that most of the reviewed studies used a poor experimental design (in particular, they lacked an active control group). We ran two experiments that used a three-group design including both an active and a passive control group, with a focus on mathematical ability. In the first experiment (*N* = 233), a group of third and fourth graders was taught chess for 25 hours and tested on mathematical problem-solving tasks. Participants also filled in a questionnaire assessing their meta-cognitive ability for mathematics problems. The group playing chess was compared to an active control group (playing checkers) and a passive control group. The three groups showed no statistically significant difference in mathematical problem-solving or metacognitive abilities in the posttest. The second experiment (*N* = 52) broadly used the same design, but the Oriental game of Go replaced checkers in the active control group. While the chess-treated group and the passive control group slightly outperformed the active control group with mathematical problem solving, the differences were not statistically significant. No differences were found with respect to metacognitive ability. These results suggest that the effects (if any) of chess instruction, when rigorously tested, are modest and that such interventions should not replace the traditional curriculum in mathematics.

Students’ poor achievement in mathematics has been the subject of debate both in the United States (Hanushek, Peterson, & Woessmann, 2012; Richland, Stigler, & Holyoak, 2012) and in Europe (Grek, 2009). Researchers and policy makers have investigated alternative methods and activities with the purpose of improving the effectiveness of mathematics teaching. One such activity is play. The rationale is that, because children are highly motivated to play, they could learn important concepts in mathematics (and other curricular domains) without realizing it, through implicit learning (Brousseau, 1997; Pelay, 2011); they could also acquire general cognitive skills such concentration and intelligence, which would positively affect their school results generally.

Several authors have argued that chess is an ideal game for educational purposes (Bart, 2014; Jerrim, Macmillan, Micklewright, Sawtell, & Wiggins, 2016; Kazemi, Yektayar, & Abad, 2012). Chess offers an optimal trade-off between complexity and simplicity, and the balance between tactics and strategy is ideal. It combines numerical, spatial, temporal, and combinatorial aspects. In addition, unlike games such as Awalé and Go, the diversity of pieces helps maintain attention—an important consideration with younger children. Altogether, these characteristics of chess may foster attention, problem solving, and self-monitoring of thinking (i.e., metacognition). Finally, there is some overlap between chess and mathematics (e.g., basic arithmetic with the value of the pieces, geometry of the board, piece movements), which is an obvious advantage when using chess to foster mathematical skills.

In recent years, considerable efforts have been made to validate these ideas empirically. Not only has chess instruction been included in the school curriculum in several countries, but several educational projects and studies involving chess are currently ongoing or have recently ended in Germany, Italy, Spain, Turkey, the United Kingdom, and the United States. Even the European Parliament has expressed its interest and positive opinion on teaching chess in schools as an educational tool (Binev, Attard-Montalto, Deva, Mauro, & Takkula, 2011). If successful, using chess in school for fostering academic achievement would shed considerable light on the question of skill acquisition and transfer (Mestre, 2005).

One psychological mechanism has been regularly proposed for explaining the putative effects of chess instruction: Being a cognitively demanding activity, chess improves pupils’ domain-general cognitive abilities (e.g., intelligence, attention, and reasoning), abilities that then transfer to other domains, and therefore benefits a wide set of non-chess-related skills (e.g., Bart, 2014). The idea is intuitive and attractive. This view of chess as a cognitive enhancer has been mentioned in popular newspapers in the United Kingdom (e.g., Garner, 2012) and was the key theoretical assumption of a recent large experimental study that took place in the United Kingdom (Jerrim et al., 2016).

## Chess skill and cognitive ability

The literature on the link between chess skill and cognitive ability is certainly consistent with this mechanism. People engaged in intellectual activities often show superior cognitive ability compared to the general population (e.g., professional musicians; Ruthsatz, Detterman, Griscom, & Cirullo, 2008), and chess is no exception. A recent meta-analysis (Sala et al. 2017) reported that chess players outperformed nonchess players in several cognitive skills (e.g., planning, numerical ability, and reasoning). The difference between the two groups was approximatively half a standard deviation. Another meta-analysis (Burgoyne et al., 2016) found positive correlations between chess skill and cognitive abilities such as fluid intelligence, processing speed, short-term and working memory (WM), and comprehension knowledge.

However, the positive relationship between chess skill and cognitive ability does not necessarily imply that chess instruction enhances cognitive ability. An alternative explanation is that individuals with better cognitive ability are more likely to excel and engage in the game of chess. To establish causality, one needs to turn attention to studies where instruction is under experimental control. This is the province of educational psychology and in particular the study of transfer of skills. This literature is rather skeptical about the possibility that an activity such as chess improves cognition generally and leads to educational benefits in topics such as mathematics. This skepticism is reinforced by the literature on expertise, which has found that experts’ knowledge is highly specialized and thus unlikely to transfer to other domains. The following section briefly summarizes these two fields of research.

## Skepticism: The question of far transfer and research into expertise

Transfer of learning occurs when a set of skills learned in one domain generalizes to one (or more) domains. It is customary to distinguish between near transfer, where transfer of learning occurs between tightly related domains (e.g., from geometry to calculus) and far transfer, where the source and target domains are only loosely related. The presumed enhancement of mathematical ability from chess instruction is a clear example of far transfer.

It has been proposed that transfer is a function of the degree to which two (or more) domains share common features (Thorndike & Woodworth, 1901). Thorndike and Woodworth’s (1901) common element theory thus predicts that while near transfer is often observed, far transfer occurs rarely. This theory has received strong support from different areas of research, where interventions that failed to obtain far-transfer effects have been documented. For example, several meta-analyses have shown that neither music instruction nor WM training enhances pupils’ cognitive ability or academic achievement (Melby-Lervåg, Redick, & Hulme, 2016; Sala & Gobet, 2017b, 2017c, in press). Interestingly, all these meta-analyses reported near-zero overall effect sizes when the treatment groups were compared to active control groups. When transfer occurs, it is almost always near transfer only. For example, Oei and Patterson (2015) have suggested that action video-game training enhances only those cognitive abilities directly involved in the particular video game used during training.

Beyond research into far transfer, research into the psychology of expertise lends support to Thorndike and Woodworth’s (1901) theory. For example, transfer is only partial between subspecialties such as cardiology and neurology (Rikers, Schmidt, & Boshuizen, 2002) and types of specialization in chess, as operationalized by the openings (first moves of a game) played (Bilalić, McLeod, & Gobet, 2009). A likely explanation is that expert performance relies substantially on perceptual information (Gobet, 2016; Gobet & Simon, 1996; Sala & Gobet, 2017a), and such information is hard to transfer to other domains. Consistent with this explanation, individuals acquire increasingly specific information as skill levels increase and, as a consequence, the probability that transfer will take place decreases considerably (Ericsson & Charness, 1994).

## Is chess special? Empirical results and the lack of an active control group

Thus, the hypothesis according to which one can improve one’s achievement in a wide set of fields by engaging in cognitively demanding activities is not supported in most areas. In fact, the abovementioned examples of music training and WM training suggest that those activities (e.g., *n*-back tasks, playing a musical instrument) do not provide any general cognitive benefit or improvement in academic achievement. Reviewing the experiments where the effects of chess instruction have been experimentally studied suggests that chess is no exception.

A recent meta-analytic review (Sala & Gobet, 2016) has evaluated the available empirical evidence regarding the effects of chess instruction on pupils’ cognitive ability and academic achievement. In that meta-analysis, the overall effect size of chess instruction was modest, with $\overline{g}$ = 0.34. It was also found that the effect sizes about measures of mathematical ability and literacy were $\overline{g}$ = 0.38 and $\overline{g}$ = 0.25, respectively. Most importantly, that review pointed out that the poor experimental design used in almost all the reviewed studies does not allow one to draw any certain conclusion about the benefits of chess instruction. In particular, most interventions did not include an active control group to control for placebo effects. Potential elements able to trigger placebo effects include the state of attention and excitement induced by a novel activity, instructors’ motivation, and teachers’ expectations. Only one study (Fried & Ginsburg, n.d.), which focused on visuospatial and perceptual abilities, included an active control group. This study showed no significant difference between the chess-treated, active, and passive control groups. Regrettably, Fried and Ginsburg’s (n.d.) experiment did not examine the effects of chess practice on pupils’ mathematical ability. Thus, that study cannot corroborate or refute any hypothesis about the effectiveness of chess instruction in enhancing mathematical ability.

Consistent with Sala and Gobet’s (2016) conclusion about the difficulty of far transfer, no effect of chess instruction was found in a recent large-scale study carried out by the Institute of Education, London, in the United Kingdom (Jerrim et al., 2016). A large sample of Year 5 pupils (9–10 years; *N* = 1,965) engaging in one year of chess instruction (ranging from 25 to 30 hours) were compared to a passive control group of peers (*N* = 1,900). The classes were randomly assigned to one of the two conditions. Pretest measures consisted of Key Stage 1 public examinations covering mathematics, science, and literacy. Posttest measures, which were obtained 1 year after the end of the treatment, consisted of Key Stage 2 public examinations in the same fields. No difference was found between the two groups in any of the measures. While some aspects of the design could have been improved (e.g., absence of an active control group, absence of measures immediately after the end of the experiment, and possible ceiling effect; Sala, Foley, & Gobet, 2017), the study certainly had strengths (e.g., large sample and allocation of classes to condition by randomization) and the absence of any positive effect of chess instruction—not even placebo effects—supports the hypothesis that far transfer is difficult.

### The present study

Given the importance of controlling for placebo effects reported in music and WM training (Melby-Lervåg et al., 2016; Sala & Gobet, 2017b, 2017c), the lack of an active control group is undoubtedly the main flaw of the studies in the field of chess instruction (Gobet & Campitelli, 2006; Gobet, de Voogt, & Retschitzki, 2004; Sala et al., 2017). The two experiments presented in this article aim to correct this unsatisfactory state of affairs. In the first experiment, primary school children receiving a 30-hour chess course were administered a test of mathematical ability and compared to both an active control group, receiving instruction about checkers, and a passive control group. Along with the test of mathematical ability, the participants were given a questionnaire assessing metacognitive abilities. Metacognitive skills have been established to be one of the most important cognitive correlates of mathematical ability (Desoete & Roeyers, 2003; Veenman, Van Hout-Wolters, & Afflerbach, 2006). Since the self-monitoring of one’s thinking processes is essential in a game like chess (De Groot, 1965), playing chess may be associated with improvements in metacognitive ability.

In the second experiment, three fourth-grade classes were randomly chosen to take part either in a chess course, a Go (Baduk) course, or regular school activities. The pupils were pre- and posttested on the same tests of mathematical ability and metacognitive ability as in the first experiment.

## Experiment 1

### Method

#### Participants

A total of 233 third and fourth graders from eight Italian schools took part in this experiment only. The mean age was 8.50 years (*SD* = 0.67 years). Parental consent was asked and obtained for all the participants.

#### Material

**Fig. 1:**
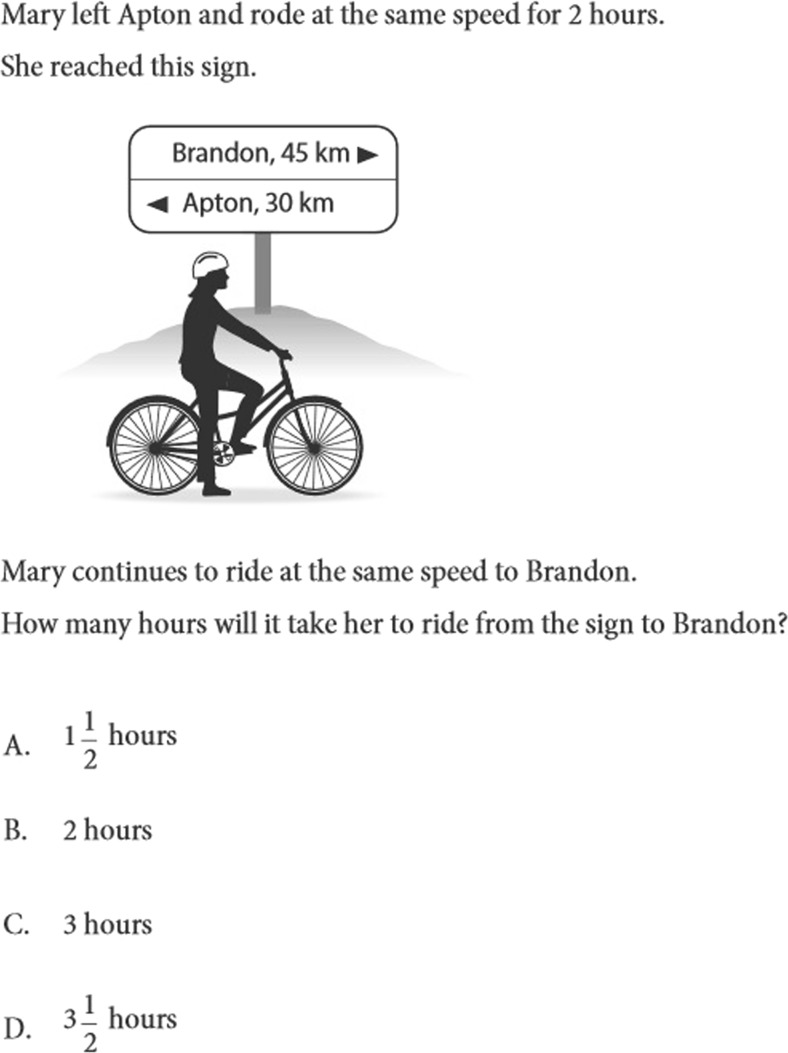
An example of the kind of problems used in the test of mathematics

A 6-item test was designed to test the pupils’ mathematical ability (range score 0–6). The items used were all from the IEA-TIMSS international survey among fourth graders (Mullis & Martin, 2013). These items were selected because they engage mathematical problem-solving ability. In fact, all the items required solving a mathematical problem starting from a given set of data. An example of the kind of mathematical problems used in IEA-TIMSS is shown in Fig. 1.

To assess participants’ metacognitive skills, we used the Italian version of Panaoura and Philippou’s (2007) questionnaire (15-item version; range score 15-75). Participants were given 45 minutes for completing the battery of tests.

#### Design

A convenience assignment to the three conditions was used. The group playing chess was compared to an active control group (playing checkers) and a passive control group (doing regular school activities). The experimental group consisted of three classes (two third-grade classes and one fourth-grade class; *N* = 53), which attended 25 hours of chess lessons during school hours,^1^ along with regular school activities. The active control group (placebo group) comprised four third-grade classes (*N* = 82), which attended 25 hours of checkers lessons during school hours, along with regular school activities. Finally, the passive control group consisted of four classes (three third-grade classes and one fourth-grade class; *N* = 98), which attended regular school activities only.

The interventions were delivered by professional instructors from the Italian Chess Federation and the Italian Checkers Federation. The chess and checkers lessons followed a prearranged teaching protocol, which consisted of the basic rules of the games, tactical exercises, and playing complete games. Most of the activities focused on problem-solving situations, such as spotting the correct move, calculating the correct variation, and evaluating the advantages/weaknesses of a position. Also, it should be noted that the two courses (chess and checkers) did not introduce any mathematics-related topics, unless these were part of the games (e.g., in chess, a Bishop is worth three Pawns).

### Results

#### Mathematical ability

A univariate analysis of covariance (ANCOVA) was used to evaluate the role of group (independent variable), mathematics pretest scores (covariate), and age (covariate), in affecting mathematics postintervention scores (dependent variable). The results showed a significant effect of pretest scores, *F*(1, 228) = 58.14, *p* < .001, and age, *F*(1, 228) = 4.22, *p* = .041, but no significant effect of group, *F*(2, 228) = 0.39, *p* = .679. The descriptive statistics are summarized in Table 1.

**Table 1:** Mathematical ability scores in the three groups (Experiment 1)

Group	Pretest	Posttest	Adjusted mean
Chess	1.75 (1.34)	1.81 (1.69)	1.64
Checkers	1.28 (0.96)	1.60 (1.14)	1.75
Control	1.41 (1.20)	1.87 (1.36)	1.83

*Note*. Standard deviations are shown in brackets

#### Metacognitive ability

The same analysis (ANCOVA) was used to analyze the results in meta-cognitive ability. The results showed a significant effect of pretest scores, *F*(1, 228) = 82.50, *p* < .001, and age, *F*(1, 228) = 3.97, *p* = .047, but no significant effect of group, *F*(2, 228) = 0.62, *p* = .541. The descriptive statistics are summarized in Table 2.

**Table 2:** Metacognitive ability scores in the three groups (Experiment 1)

Group	Pretest	Posttest	Adjusted mean
Chess	54.19 (9.76)	52.92 (8.86)	52.35
Checkers	54.51 (7.39)	55.07 (8.81)	53.86
Control	51.41 (9.09)	52.22 (9.71)	53.55

*Note*. Standard deviations are shown in brackets

### Discussion

The results showed no significant differences between the three groups in mathematical ability or metacognitive ability.

## Experiment 2

The second experiment^2^ broadly used the same design but also differed in three ways. First, the classes were randomly assigned to the experimental conditions. Second, the active control group played the Oriental game of Go (Baduk) instead of checkers. Finally, chess and Go replaced part of the hours (*n* = 15) originally dedicated to mathematics and sciences to directly compare the two games with the traditional methods of teaching mathematics and mathematics-related disciplines.

### Method

#### Participants

Fifty-two fourth graders in three classes of a primary school in Italy took part in this experiment. The mean age of the participants was 9.32 years (*SD* = 0.32 years). Parental consent was asked and obtained for all the participants.

#### Material

The same tests as those used in Experiment 1 were administered to the participants.

#### Design

The three classes were randomly assigned to three groups. The first class attended 15 hours of chess lessons during school hours, along with regular school activities (experimental group). The second class attended regular school activities only (passive control group). Finally, the third class attended 15 hours of Go lessons during school hours, along with regular school activities (active control/placebo group).

Importantly, the two interventions—that is, chess and Go courses—substituted part of the hours originally devoted to mathematics and sciences. This way, we could compare the effectiveness of chess (and Go) instruction with the traditional didactics of teaching mathematics and mathematics-related disciplines, such as sciences. Like in Experiment 1, the chess and Go lessons followed a prearranged teaching protocol. To rule out possible effects related to instructor behavior (e.g., Pygmalion effect), the chess and Go interventions were delivered by the same instructor, who was both a chess and Go trainer. The participants were pre- and posttested on mathematical ability and metacognition, once before the beginning of the intervention and once after the end.

### Results

#### Mathematical ability

No significant differences between the three groups were found in the pre-test scores, *F*(2, 51) = 1.03, *p* = .365. A univariate analysis of covariance (ANCOVA) was used to evaluate the role of group (independent variable) and mathematics pretest scores (covariate) in affecting mathematics postintervention scores (dependent variable). The results showed a significant effect of the covariate, *F*(1, 48) = 21.83, *p* < .001, and a significant effect of group, *F*(2, 48) = 3.37, *p* = .043. The pairwise comparisons showed that the control group outperformed the Go group (*p* = .017), the chess group marginally outperformed the Go group (*p* = .088), whereas no significant difference was found between the control and the chess group (*p* = .487). A more conservative post hoc analysis (Bonferroni correction) showed only a marginal difference between the control group and the Go group (*p* = .052). No other significant difference was found. The descriptive statistics are summarized in Table 3.

**Table 3:** Mathematical ability scores in the three groups (Experiment 2)

Group	Pretest	Posttest	Adjusted mean
Chess	2.13 (1.26)	2.50 (1.41)	2.30
Go	1.81 (1.08)	1.62 (1.20)	1.63
Control	1.53 (1.13)	2.40 (1.55)	2.60

*Note*. Standard deviations are shown in brackets

#### Metacognitive skills

No significant differences between the three groups were found in the pretest scores, *F*(2, 51) = 0.49, *p* = .617. A univariate analysis of covariance (ANCOVA) was used to evaluate the role of group (independent variable) and metacognition pretest scores (covariate) in affecting metacognition postintervention scores (dependent variable). The results showed a significant effect of the covariate, *F*(1, 48) = 47.81, *p* < .001, and no significant effect of group, *F*(2, 48) = 0.37, *p* = .694. The pairwise comparisons showed no differences between the three groups. The descriptive statistics are summarized in Table 4.

**Table 4:** Metacognitive skill scores in the three groups (Experiment 2)

Group	Pretest	Posttest	Adjusted mean
Chess	55.2 (11.0)	57.0 (10.5)	56.3
Go	52.7 (9.2)	54.8 (8.6)	55.8
Control	55.3 (6.5)	58.3 (6.0)	57.6

*Note*. Standard deviations are shown in brackets

#### Discussion

The effects of chess instruction on mathematical problem-solving ability were minimal. Children seemed to benefit more from the traditional didactics than from chess and Go instruction. Regarding metacognitive skills, children did not seem to benefit from any advantage from the 15-hour chess course. In fact, the participants performed equally across the three groups, suggesting that metacognition does not represent the cognitive link between chess instruction and mathematical ability.

## General discussion

The results of the two studies do not support the hypothesis according to which chess instruction benefits pupils’ mathematical ability. The effects of chess, if any, appear to be minimal and certainly too limited to provide any educational advantage over the traditional instructional methods. Thus, chess instruction seems to align with the results obtained in the fields of music instruction and WM training. In a broader perspective, our findings are in line with Thorndike and Woodworth’s (1901) common element theory and substantial research on expertise (Gobet, 2016) and education (Donovan, Bransford, & Pellegrino, 1999) in predicting no far-transfer effects.

### Recommendations for future research

Given the small number of studies controlling for placebo effects, it is imperative to replicate and extend the experiments reported in the present article. Compared to the design we adopted, examples of possible ameliorations include full random assignment to the groups, measures of other cognitive constructs (e.g., intelligence and spatial cognition), and the manipulation of the duration of the chess interventions.

**Fig. 2:**
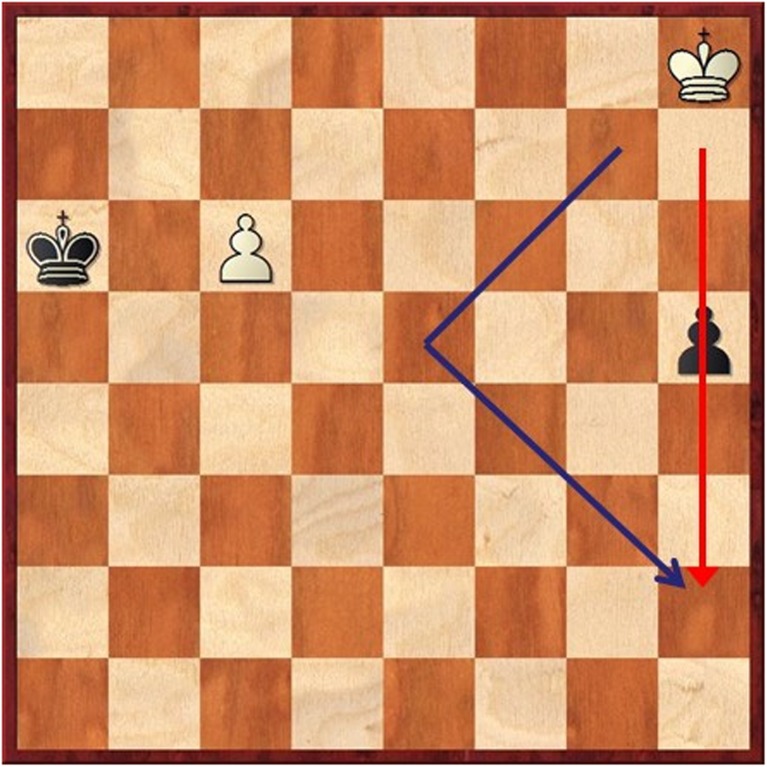
Using chess to illustrate block-city distance and Euclidean distance. White draws the game by moving the King along the blue line, which allows him both to approach his Pawn (threatening promotion) and to catch the black Pawn. In chess, block city and Euclidean distances are equivalent (in this examples, six moves in both cases to reach the square where the two arrows meet). This position was composed by Richard Réti in 1921

In addition, an interesting way to make chess instruction more effective could be to make links between mathematics and chess explicit. Possible examples comprise introducing the Cartesian graph to pupils with the chess board and illustrating the concept of block distance—as opposed to distance in Euclidean space—with the movement of the King (see Fig. 2). The inclusion of domain-specific information (e.g., mathematical problems) into chess courses curricula may be a simple way to get around the limits of far transfer to occur. One variation of this approach is to use not only chess but also other board games or even other types of games such as card games to teach specific mathematical concepts. For example, mancala games could be used for teaching the concept of modular arithmetic, card games for teaching elements of probability, and Nim games to teach the binary system of Boolean algebra (Rougetet, 2016).

## Conclusion

Beyond chess, the results of the research on chess instruction have profound implications for our understanding of learning and transfer of skill. There is a stark contrast between the enthusiasm displayed by the chess community and the sobering results from research on transfer and expertise: While the former heralds the positive benefits of chess instruction, the latter consistently report data speaking against the occurrence of far transfer. When critically evaluated, the literature on chess instruction is consistent with other experimental studies on transfer, indicating that far transfer is very unlikely. The results of the two experiments presented in this paper are consistent with these conclusions.

Extrapolating from the research on chess and activities such as music and video-game playing, it is likely that the same difficulties in far transfer will be found with other kinds of games and play. To make the use of didactical games more effective, and given the difficulty of far transfer to occur, teachers and researchers should seriously consider the possibility of making explicit the link between playing games and the mathematical abilities the game is supposed to foster. Even so, it is worth reminding ourselves of French sociologist Roger Caillois’s (1957) discussion of the role of play in his article on the unity of play and diversity of games: “Faculties thus developed certainly profit by this supplementary training which is free, intense, pleasurable, inventive, and secure. But it is never the function of play itself to develop these faculties. The purpose of play is play” (p. 105).

## Footnotes

1 The chess and checkers courses were implemented during school hours accordingly to the teachers’ availability. No particular discipline (e.g., mathematics) was systematically replaced by the courses.

2 The results of this experiment were published in Sala, Gobet, Trinchero, and Ventura (2016).
